# *Drosophila* Ana1 is required for centrosome assembly and centriole elongation

**DOI:** 10.1242/jcs.186460

**Published:** 2016-07-01

**Authors:** Saroj Saurya, Hélio Roque, Zsofia A. Novak, Alan Wainman, Mustafa G. Aydogan, Adam Volanakis, Boris Sieber, David Miguel Susano Pinto, Jordan W. Raff

**Affiliations:** 1 Sir William Dunn School of Pathology, University of Oxford, South Parks Road, Oxford OX1 3RE, UK; 2 Micron Oxford Advanced Bioimaging Unit, Department of Biochemistry, University of Oxford, South Parks Road, Oxford OX1 3QU, UK

**Keywords:** Centriole, Centrosome, Ana1, Cep295, Centriole elongation, Centriole structure

## Abstract

Centrioles organise centrosomes and cilia, and these organelles have an important role in many cell processes. In flies, the centriole protein Ana1 is required for the assembly of functional centrosomes and cilia. It has recently been shown that Cep135 (also known as Bld10) initially recruits Ana1 to newly formed centrioles, and that Ana1 then recruits Asl (known as Cep152 in mammals) to promote the conversion of these centrioles into centrosomes. Here, we show that *ana1* mutants lack detectable centrosomes *in vivo*, that Ana1 is irreversibly incorporated into centrioles during their assembly and appears to play a more important role in maintaining Asl at centrioles than in initially recruiting Asl to centrioles. Unexpectedly, we also find that Ana1 promotes centriole elongation in a dose-dependent manner: centrioles are shorter when Ana1 dosage is reduced and are longer when Ana1 is overexpressed. This latter function of Ana1 appears to be distinct from its role in centrosome and cilium function, as a GFP–Ana1 fusion lacking the N-terminal 639 amino acids of the protein can support centrosome assembly and cilium function but cannot promote centriole over-elongation when overexpressed.

## Abstract (precis)

**Highlighted Article:** Ana1 is a conserved centriole protein that we show is required for centrosome and cilium assembly and that also helps to promote centriole elongation in a dose-dependent manner.

## INTRODUCTION

Centrioles are ancient cellular organelles that are required for the formation of centrosomes and cilia (Azimzadeh, 2014). These organelles play an important role in many cell processes, and their dysfunction has been linked to a diverse set of human pathologies (Bettencourt-Dias et al., 2011; Conduit et al., 2015a; Nigg and Raff, 2009). A better understanding of how these organelles are assembled is therefore an important goal of modern cell biology.

Recent studies have revealed that only a relatively small number of proteins are essential for centriole assembly (Conduit et al., 2015a; Fırat-Karalar and Stearns, 2014; Gönczy, 2012; Jana et al., 2014). These proteins form a conserved pathway in which the protein kinase ZYG-1 (in worms), Plk4 (in mammals) or Sak (in flies) (Bettencourt-Dias et al., 2005; Delattre et al., 2006; Habedanck et al., 2005; Pelletier et al., 2006) recruits SAS-5/Ana2/STIL (worms, flies and mammals, respectively) and Sas-6 to the side of the mother centriole to form a cartwheel structure that initiates the assembly of the new daughter centriole (Arquint et al., 2015; Dzhindzhev et al., 2014; Kitagawa et al., 2011; Kratz et al., 2015; Lettman et al., 2013; Moyer et al., 2015; Ohta et al., 2014; Qiao et al., 2012; Stevens et al., 2010b; van Breugel et al., 2011). These proteins then recruit Sas-4/CPAP (CPAP is also known as CENPJ), which in turn helps recruit the centriolar microtubules (Cottee et al., 2013; Hatzopoulos et al., 2013; Hsu et al., 2008; Pelletier et al., 2006; Tang et al., 2011). In worms, SPD-2 recruits ZYG-1 to the mother centrioles (Delattre et al., 2006; Pelletier et al., 2006), whereas this function is performed by Asterless (Asl) in flies (Dzhindzhev et al., 2010; Novak et al., 2014). Vertebrate cells appear to use a combination of these proteins (Cep192 and Cep152, respectively) (Kim et al., 2013; Park et al., 2014; Sonnen et al., 2013).

Genome-wide RNA interference (RNAi) screens in flies have identified Ana1 as a protein that is potentially required for centriole assembly (Dobbelaere et al., 2008; Goshima et al., 2007). Flies mutant for *ana1* are uncoordinated (a phenotype indicative of cilia defects) and exhibit reduced centrosome numbers in larval brain cells (Blachon et al., 2009), strongly suggesting that Ana1 has a role in centriole, centrosome and cilium assembly. A recent study on the human Ana1 homologue (Cep295) has suggested that this protein is not essential for centriole assembly, but rather is required for centriole-to-centrosome conversion (Izquierdo et al., 2014). New centrioles were not converted into centrosomes in the absence of Cep295 and so could not organise their pericentriolar material (PCM) properly: as a result, the new centrioles appeared to be destabilised once they lost their central cartwheel (as occurs normally in most vertebrate cells when the daughter centrioles are converted into mothers). Thus, vertebrate centrioles appear to be stabilised by both the central cartwheel and the PCM they organise. If newly formed centrioles cannot be converted into centrosomes (and so cannot organise any PCM) they are destabilised once they lose their central cartwheel – potentially explaining why Ana1/Cep295 proteins might be essential for both centrosome and cilium assembly. Like Cep295 in human cells, Ana1 appears to be required for centriole-to-centrosome conversion in fly cells (Fu et al., 2016). Ana1 is first recruited to centrioles by Cep135 (also known as Bld10) in late interphase and Ana1 subsequently recruits Asl to centrioles during mitosis (Fu et al., 2016). Asl plays a particularly important part in centriole-to-centrosome conversion in flies, as its incorporation is required to allow new centrioles to duplicate (Novak et al., 2014) and to recruit mitotic PCM for the first time (Conduit et al., 2014b).

Here, we investigated the function of Ana1 in flies *in vivo*. We show that *ana1* mutant flies have very few centrosomes, and that Ana1 appears to be irreversibly incorporated into centrioles throughout S-phase with unusual dynamics. A structure–function analysis suggests that the recruitment of Ana1 to centrioles, and the role of Ana1 in recruiting Asl to centrioles, might be more complicated than previously thought. Unexpectedly, we find that Ana1 also promotes centriole elongation in a dose-dependent manner, and this function appears to be mechanistically different to its role in promoting Asl recruitment, and centrosome and cilium assembly.

## RESULTS

### Fly tissues lacking Ana1 protein have very few centrosomes

**Fig. 1.**
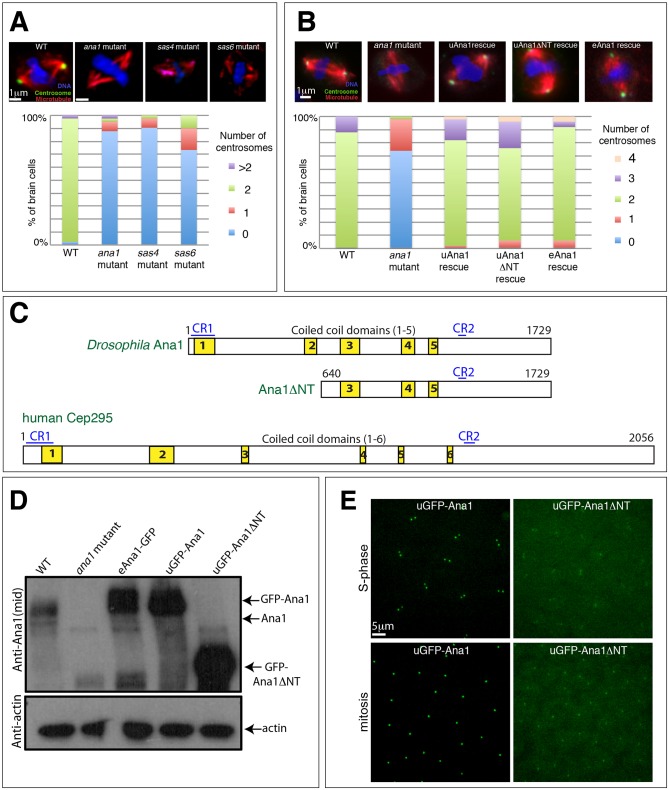
**Ana1 is required for efficient centrosome assembly and/or maintenance.** (A,B) Micrographs showing images of third-instar larval brain cells stained for DNA (blue), microtubules (red) and the centrosome marker Asl (green) in WT, various mutants (as indicated), or the *ana1* mutant rescued by different transgenes (as indicated). Graphs quantify the percentage of cells of each genotype exhibiting different numbers of centrosomes (*n*=30–50 total cells counted from five brains). Centrosomes were scored as dots in mitotic cells that were positive for both the centriole and centrosome protein Asl and the centrosome protein Cnn (Conduit et al., 2014a). (C) A schematic representation of *Drosophila* Ana1, Ana1ΔNT and human Cep295 proteins. Two conserved regions (CR1 and CR2, blue lines) and several predicted coiled-coil domains (yellow boxes) are indicated. (D) Western blots of *Drosophila* third-instar larval brains from WT, *ana1* mutant or WT brains expressing Ana1–GFP from its endogenous promoter (eAna1–GFP), or GFP–Ana1 or GFP-Ana1-ΔNT from the ubiquitin promoter (uGFP–Ana1 or uGFP–Ana1ΔNT). Blots were probed with anti-Ana1 or anti-actin antibodies (loading control). Serial dilution blots (not shown) reveal that the GFP fusions are overexpressed relative to endogenous Ana1 by ∼2–4× (eAna1–GFP), 3–5× (uGFP–Ana1) or 5–10× (uGFP–Ana1ΔNT). (E) Micrographs showing representative images of *Drosophila* embryos expressing uGFP–Ana1 (left panels) or uGFP–Ana1ΔNT (right panels) in a WT background in S-phase (top panels) or in mitosis (bottom panels).

It has been shown previously that Ana1 has a role in centriole, centrosome and cilium formation (Blachon et al., 2009; Dobbelaere et al., 2008; Goshima et al., 2007), and *ana1* mutant flies are severely uncoordinated due to the lack of functional cilia (Blachon et al., 2009). We found that *ana1* mutants have a dramatic reduction in centrosome numbers in third-instar larval brains that is comparable to that observed in brains mutant for the essential centriole assembly genes *Sas-4*, *Sas-6*, *ana2* and *asl* (Fig. 1A) (Basto et al., 2006; Baumbach et al., 2015; Cottee et al., 2015; Peel et al., 2007; Rodrigues-Martins et al., 2007). Thus, Ana1 has an important role in centrosome assembly *in vivo*.

### The N-terminal region of Ana1 is not essential for centrosome assembly or cilium function

At the time we initiated our studies, the *ana1* gene was thought to encode two polypeptides – a long form and a shorter form that lacks the N-terminal 639 amino acids (Fig. 1C) (Blachon et al., 2009). The larger protein has two short conserved regions – an N-terminal region denoted CR1 and a more C-terminal region denoted CR2 – and an extended middle region comprising several predicted coiled-coils. The shorter form is missing CR1 and the first two predicted coiled-coils. The most recent release of Flybase, however, indicates that only the long polypeptide is produced *in vivo*, so we refer to the long-form as wild-type (WT) Ana1 and the short form as N-terminally deleted Ana1 [Ana1ΔNT(640–1729), hereafter Ana1ΔNT]. We obtained a transgenic line expressing WT Ana1–GFP from its endogenous promoter (eAna1–GFP) (Blachon et al., 2009), and we generated transgenic lines expressing WT GFP–Ana1 or GFP–Ana1ΔNT from the Ubiquitin promoter (uGFP–Ana1 or uGFP–Ana1ΔNT, respectively) (Stevens et al., 2010a).

Western blotting revealed that all these fusion proteins (including the one driven by the endogenous promoter) were overexpressed compared to the endogenous protein (Fig. 1D). Surprisingly, both GFP–Ana1 and GFP–Ana1ΔNT strongly rescued the severe uncoordination defect of *ana1* mutant flies to similar extents (data not shown), but mutant flies rescued by GFP–Ana1 were fertile, whereas mutant flies rescued by GFP–Ana1ΔNT were both male- and female-sterile. Moreover, both fusion proteins appeared to support centrosome assembly in third-instar larval brains to similar extents (Fig. 1B), and they both were recruited to centrosomes when expressed in embryos, although the centrosomal localisation of GFP–Ana1ΔNT was markedly reduced compared to GFP–Ana1 (Fig. 1E; see below). Taken together, these results suggest that the N-terminal 639 amino acids of Ana1 are not essential for centrosome assembly and cilium function, although flies surviving on the GFP–Ana1ΔNT fusion protein are male- and female-sterile.

### A structure–function analysis of Ana1

**Fig. 2.**
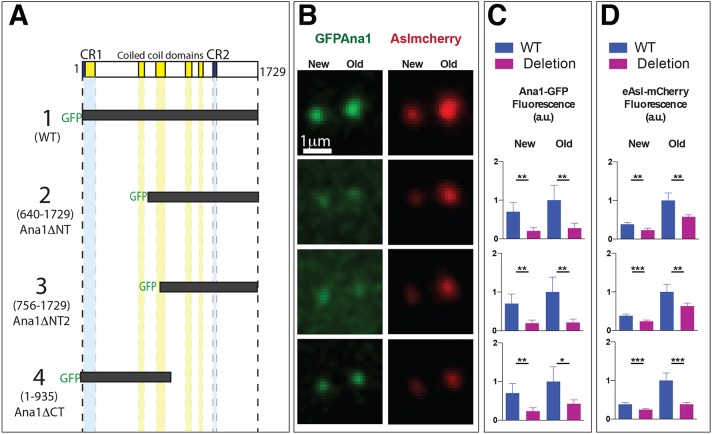
**A structure–function analysis of Ana1.** (A) A schematic representation of the various GFP–Ana1 deletion constructs analysed. *In-vitro*-transcribed mRNA encoding each of these constructs was injected into WT embryos expressing eAsl–mCherry; the distribution of each fusion protein was analysed in living embryos in mid-S-phase (as judged by the separation of the centrosomes in the embryo). (B) Micrographs showing the typical localisation of each GFP–Ana1 fusion protein (green) and Asl–mCherry (red) at new and old centrosomes in embryos injected with the Ana1 constructs as shown in A. (C,D) The bar graphs show the mean±s.d. centrosomal Ana1–GFP fusion protein (C) or Asl–mCherry (D) fluorescence at new and old centrosomes. All values were normalised to the average fluorescence level of WT GFP–Ana1 or Asl–mCherry observed at old centrosomes in contemporaneous controls injected with WT GFP–Ana1 (construct 1) (arbitrarily given a value of 1). Note that the preferential asymmetric localisation of Asl–mCherry to the old centrosome was consistently reduced in embryos expressing Ana1ΔCT (construct 4). *n*=5 embryos for each construct. *P<0.05; **P<0.001; ***P<0.0001 (paired two-tailed *t*-test).

The finding that GFP–Ana1ΔNT can support centrosome assembly *in vivo* is perhaps surprising, as a recent study has suggested that, in cultured cells, the N-terminal region of Ana1 (amino acids 1–935) is required to target Ana1 to centrioles through a direct interaction with Cep135, whereas the C-terminal region of Ana1 (amino acids 756–1729) interacts with Asl, but is not targeted to centrioles (even in the presence of the endogenous protein) (Fu et al., 2016). To analyse the potential function of the various regions of Ana1 in embryos in more detail, we synthesised mRNAs encoding GFP–Ana1, GFP–Ana1ΔNT or the N-terminal or C-terminal deletions analysed by Fu et al. (GFP–Ana1ΔNT2 or GFP–Ana1ΔCT, respectively) (Fig. 2A). We injected these into WT embryos (that therefore contain endogenous unlabelled Ana1) expressing Asl–mCherry; the injected mRNAs are then gradually translated and can compete for binding partners with the endogenous protein (Cottee et al., 2015; Richens et al., 2015). We then analysed the behaviour of the expressed proteins and their effect on Asl–mCherry localisation at 60–90 min after injection (Fig. 2B–D).

Full-length GFP–Ana1 strongly localised to centrosomes and had no discernible effect on the centrosomal localisation of Asl–mCherry, which is preferentially localised at ‘old’ centrosomes (that contain the older mother centrioles) because the ‘new’ centrosomes (that contain the younger mother centrioles) only start to recruit Asl at around the time the young mother centrioles separate from their mothers (Novak et al., 2014) (Fig. 2, construct 1). We noticed that GFP–Ana1 also often exhibited a slight asymmetry and was often slightly enriched on the old centrosome (see below). GFP–Ana1ΔNT and GFP–Ana1ΔNT2 both localised to centrioles, although much more weakly than WT GFP–Ana1 (Fig. 2, constructs 2 and 3). Moreover, the amount of Asl–mCherry localised to centrosomes was also significantly reduced. This is perhaps surprising because the C-terminal region of Ana1 is thought to recruit Asl to centrioles (Fu et al., 2016), so it is unclear why recruiting a C-terminal fragment of Ana1 to centrosomes should perturb Asl levels. Perhaps the cytoplasmic fractions of these proteins sequester cytoplasmic Asl–mCherry, preventing its recruitment to centrosomes.

Interestingly, GFP–Ana1ΔCT also exhibited a markedly reduced localisation to centrosomes, although its localisation appeared to be tighter and less diffuse than that observed with GFP–Ana1ΔNT or GFP–Ana1ΔNT2 (Fig. 2, construct 4). This reduction in centrosomal localisation is also perhaps surprising as GFP–Ana1ΔCT contains the entire N-terminal domain that interacts with Cep135, and that domain is both necessary and sufficient to recruit Ana1 to centrosomes in cultured cells (Fu et al., 2016). Moreover, although GFP–Ana1ΔCT expression strongly perturbed the centrosomal localisation of Asl–mCherry (as expected because GFP–Ana1ΔCT should not be able to recruit Asl), it more strongly affected the localisation at the old centrosome than at the new centrosome. As a result, the preferential accumulation of Asl at the old centrosome was greatly reduced: the ratio of Asl–mCherry fluorescence at old versus young centrosomes was 2.6±0.3; 2.6±0.5 and 2.7±0.3 (mean±s.d., *n*=5 embryos, 10 centrosomes per embryo) in embryos expressing WT GFP–Ana1, GFP–Ana1ΔNT and GFP–Ana1ΔNT2, respectively, but only 1.5±0.1 in embryos expressing GFP–Ana1ΔCT (*P*<0.001; two tailed *t*-test). Thus, most surprisingly, GFP–Ana1ΔCT preferentially perturbs the localisation of Asl–mCherry at old centrosomes, rather than at the recently converted new centrosomes (see Discussion).

### Overexpressing Sas-6 and Ana2 can stabilise centrioles in the absence of Ana1

**Fig. 3.**
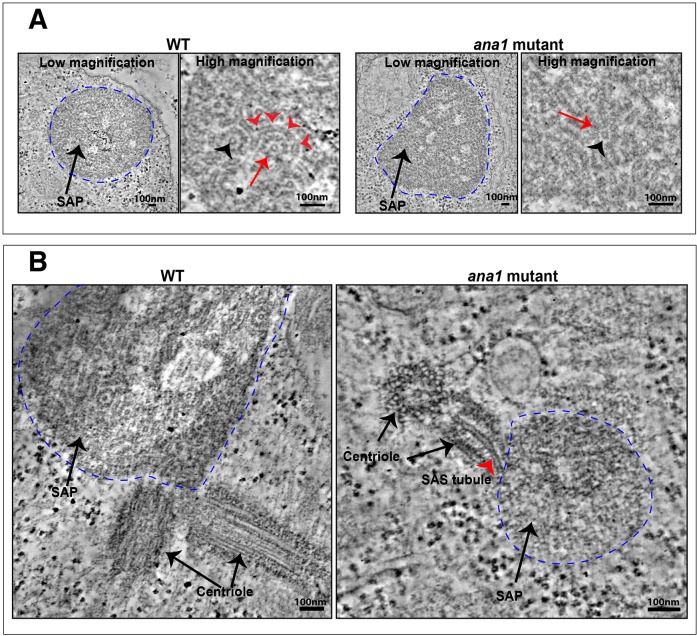
**Ana1 is not essential for SAP assembly.** (A) Micrographs show low- and high-magnification views from electron microscopy tomographs of a SAP particle (dotted blue line) in a WT (left panels) or *ana1* mutant (right panels) spermatocyte. The SAP in the WT cell is highly structured and consists of well-organised ‘SAStubules’ that resemble the central cartwheel of the centriole (Stevens et al., 2010b). An end-on view of a SAStubule is highlighted by the black arrowhead, and a transverse view is highlighted with red arrowheads. The SAStubules are linked by spokes to an electron-dense outer ring (red arrow). In *ana1* mutants, the SAP structures are less well-defined, but the central cartwheel-like SAStubules are still easily discerned (red arrow), especially in an end-on view (black arrowhead). (B) The SAPs in WT spermatocytes are often directly connected to the centrioles, and we could detect small centriole-like structures associated with the SAPs in *ana1* mutants in seven out of 11 of the SAPs we examined: in this example the central cartwheel of one of the centriole-like structures appears to be directly connected to a SAStubule that extends into the SAP (red arrowhead).

Ana1 is required for both centrosome and cilium function in flies, suggesting that it likely plays some part in centriole assembly. We wanted to test whether Ana1 might have a role in assembling and/or stabilising the centriole central cartwheel structure. We have previously shown that the co-overexpression of Sas-6 and Ana2 in spermatocytes leads to the assembly of Sas-6 and Ana2 particles (SAPs) that are composed of long tubules (SAStubules) that resemble the central cartwheel (Stevens et al., 2010b). We therefore overexpressed Sas-6 and Ana2 in *ana1* mutant spermatocytes. SAPs still formed in these cells, but these were less abundant and larger than normal (Fig. S1). Electron microscopy tomography revealed that the SAPs were generally less well-ordered, but structures resembling the cartwheel central hub were still clearly visible (black arrowheads, Fig. 3A). Thus, SAP structure and behaviour is altered in the absence of Ana1 – supporting the idea that Ana1 influences centriole structure – but Ana1 does not appear to be essential for the assembly of a central cartwheel-like structure.

Surprisingly, we noticed that structures resembling centrioles that were closely associated with the SAPs were detected in seven of the 11 of the *ana1* mutant spermatocytes we examined, suggesting that centriole-like structures can still assemble in these spermatocytes under certain conditions (Fig. 3B). Interestingly, in one of these structures, we observed the central cartwheel of the centriole-like structure directly connected to a SAStubule emanating from the SAP (red arrowhead, Fig. 3B), supporting our conclusion that these tubules are closely related to central cartwheels. The centriole-like structures associated with the SAPs in *ana1* mutant cells appeared to be much shorter than the very large centrioles normally found in spermatocytes, indicating that Ana1 has a role in promoting centriole elongation in these cells (see below).

### Ana1 is oriented within the centriole and is irreversibly incorporated into daughter centrioles as they assemble in S-phase

**Fig. 4.**
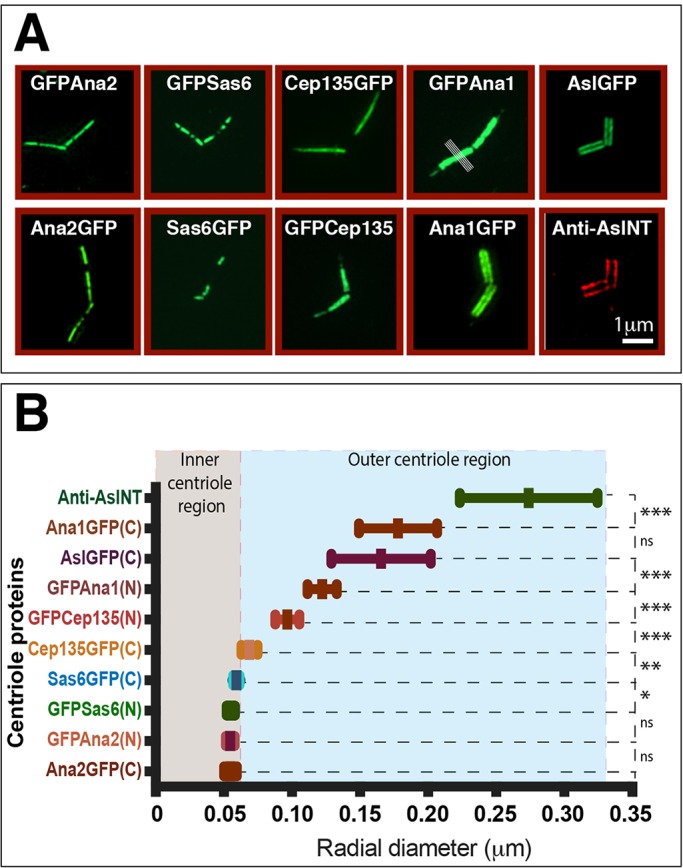
**Ana1, Cep135 and Asl molecules are oriented within spermatocyte centrioles.** (A) Micrographs showing 3D-SIM images of centrioles in primary spermatocytes expressing various centriole proteins tagged with GFP at either their N- or C-termini and stained for GFP (green). The bottom-right image shows a centriole stained with anti-Asl-NT antibodies (red). The white lines in the GFPAna1 panel indicate example lines drawn through the centriole to calculate a fluorescence intensity distribution. (B) Graph showing the mean±s.d. radial diameter of each centriole marker (as indicated, see Materials and Methods) (*n*=10–53 centrioles). Statistical significance of the different radial diameters between successive proteins – moving from the innermost (Ana2–GFP) to the outermost (Asl-NT) – was calculated using a paired two-tailed *t*-test. *P<0.05; **P<0.001; ***P<0.0001; ns, not significant. Note that the radial diameters of the N-terminal region of Cep135 and the N-terminal region of Ana1 – which can directly interact with one another *in vitro* – are significantly different.

To gain insight into how Ana1 might function at centrioles, we used three-dimensional (3D) structured illumination super-resolution microscopy (3D-SIM) to investigate its localisation relative to other centriole proteins in the large centrioles found in *Drosophila* spermatocytes (Fig. 4A,B). We expressed N- or C-terminally tagged GFP fusion proteins to several centriole proteins in WT spermatocytes, and measured an average fluorescence intensity profile across ten lines drawn through each spermatocyte centriole (as indicated by the grill-like white lines in the GFP–Ana1 panel in Fig. 4A). This allowed us to calculate an average radial diameter for each protein (see Materials and Methods). As reported previously from studies on centrosomes in cultured fly cells, Cep135, Ana1 and Asl molecules were all asymmetrically localised within the centriole (Fu and Glover, 2012; Fu et al., 2016; Mennella et al., 2012) (Fig. 4A,B). All of these molecules were localised outside the cartwheel region occupied by Sas-6 and Ana2 (a region that is below the resolution of our 3D-SIM system). As in cultured cell centrosomes, the C-terminal regions of Ana1 and Asl – which directly interact *in vitro* – were essentially overlapping in the spermatocyte centrioles. In contrast to cultured cell centrosomes, however, the N-terminal regions of Cep135 and Ana1 – which also directly interact *in vitro* – were close to each other, but were essentially non-overlapping in the spermatocyte centrioles. As these studies report the localisation of the GFP moiety (rather than the actual N-termini of these proteins), it is difficult to interpret the importance of this observation.

**Fig. 5.**
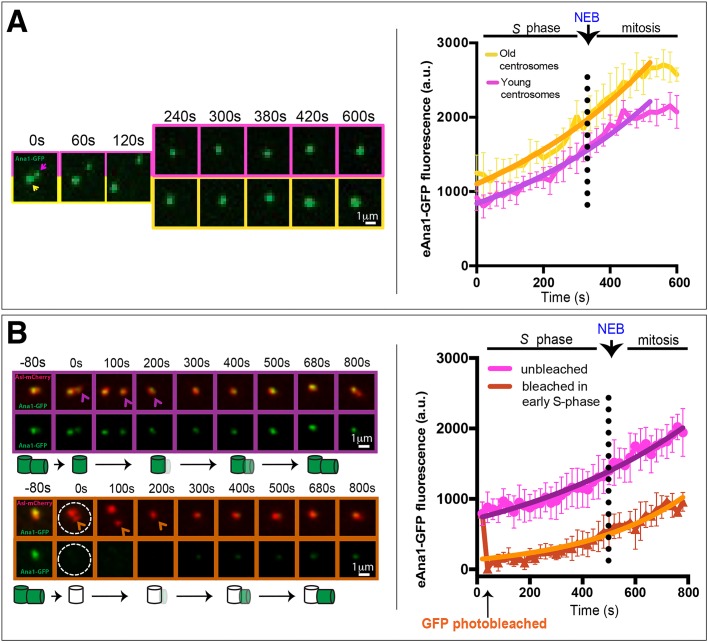
**Ana1 is stably incorporated into centrioles during S-phase and mitosis.** (A) Micrographs showing images from a time-lapse movie following eAna1–GFP incorporation into newly separated centrosomes. The graphs show the mean±s.d. centrosomal eAna1–GFP fluorescence over time averaged from three embryos (10 centrosomes per embryo). Time (s) relative to centriole separation (*t*=0) is indicated. Old (yellow arrow, boxes and line on graph) and young centrosomes (purple arrow, boxes and line on graph) were measured independently. Non-linear regression analysis indicates that the pattern of Ana1–GFP incorporation is best fitted by an exponential function (solid line), with incorporation plateauing in late mitosis. (B) Micrographs showing images from a time-lapse movie of a FRAP experiment following GFP–Ana1 incorporation in control (purple arrow, boxes and line on graph) and photobleached (orange arrow, boxes and line on graph) centrosomes (both taken at the same time form the same embryo). A schematic of the interpretation of these results is shown beneath the images. The graphs show the mean±s.d. eAna1–GFP fluorescence over time averaged from three embryos (six photobleached or non-photobleached centrosomes per embryo). These embryos also expressed eAsl–mCherry (red) so that centrioles could be followed after photobleaching. Note that after the 100 s timepoint only the dimmer (younger) centrosome is shown in the micrographs. Time (s) relative to centriole photobleaching (which was performed at centriole separation, *t*=0) is indicated.

We next examined how Ana1 incorporates into centrioles by following the dynamic behaviour of Ana1 in the early fly embryo. In these embryos, the nuclei exist in a common cytoplasm (the syncytium) and proceed nearly synchronously through rapid cycles of alternating S- and M-phases without intervening gap phases. Each mother centriole forms a daughter centriole early in S-phase that grows and reaches its full length by M-phase (Callaini and Riparbelli, 1990). We have previously shown that Sas-4 is incorporated at an approximately linear rate into the growing daughter centrioles throughout S-phase in these embryos (Conduit et al., 2015b; Novak et al., 2014), whereas Asl is not incorporated into the new daughter centriole during S-phase, but starts incorporating into new centrioles at an approximately linear rate when they separate from their mothers during mitosis (Novak et al., 2014). As GFP–Ana1 is slightly enriched on old centrosomes (Fig. 2C), we measured the incorporation profile of GFP–Ana1 at old (yellow boxes and lines, Fig. 5A) and new (purple boxes and lines, Fig. 5A) centrosomes independently. GFP–Ana1 exhibited an ‘exponential’ incorporation profile at both centrosomes, that is, incorporating at a rate that was low in early S-phase, but that gradually increased throughout S-phase and into mitosis, before plateauing in late mitosis (Fig. 5A). Clearly, more work will be required to understand the importance of this unexpected incorporation profile.

To test whether Ana1 is incorporated irreversibly into centrioles, we performed a fluorescence recovery after photobleaching (FRAP) analysis (Fig. 5B). We found that centrioles bleached in early S-phase exhibited a very similar incorporation profiled to non-bleached centrioles, strongly suggesting that the fluorescence recovery we observe is primarily driven by incorporation into the new daughter centrioles and that there is very little turnover of GFP–Ana1 at the mother centriole (see schematic interpretation, Fig. 5B). Thus, Ana1 appears to be stably incorporated into centrioles during their assembly. This might explain why GFP–Ana1 appears to be slightly enriched on old centrosomes: as GFP takes some time to mature and fluoresce, older centrioles will contain a higher proportion of fluorescent GFP–Ana1 than newly formed centrioles. A similar phenomenon has been reported for GFP–PACT, a centriole protein that is also irreversibly incorporated into centrioles (Conduit et al., 2010).

### Ana1 levels influence centriole length, and this requires its N-terminal region

**Fig. 6.**
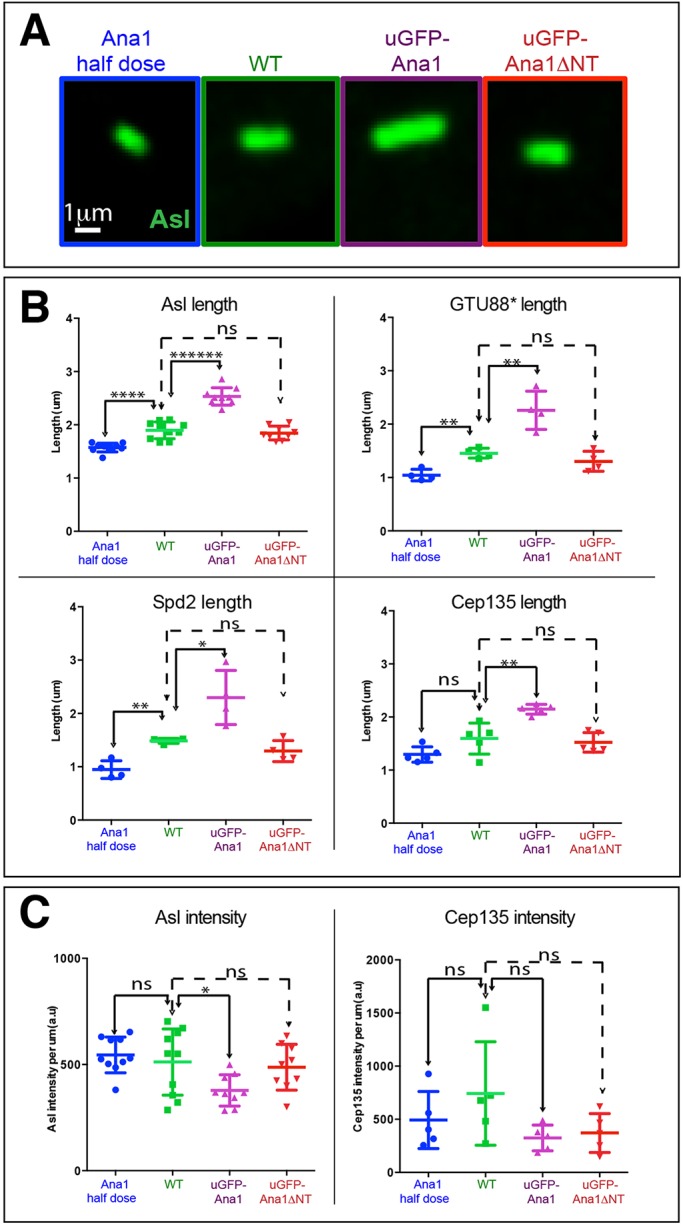
**Ana1 levels influence centriole length.** (A) Micrographs showing typical examples of meiosis II centrioles (revealed by Asl staining) in pupal testes from *ana1* heterozygous mutants (containing only one copy of the WT gene), WT, WT overexpressing GFP–Ana1 and WT overexpressing GFP–Ana1ΔNT. (B) Graphs show the mean±s.d. quantification of meiosis II centriole length using four different centriole markers for each genotype. (C) Graphs show the mean±s.d. quantification of Asl or Cep135 fluorescence intensity (per unit length of centriole) for each genotype. Each point on the graphs represents the average length or fluorescence intensity measured from a single testis, and 20–40 centrioles were measured in each testis. *P<0.05; **P<0.001; ****P<0.00001; ******P<0.0000001; ns, not significant (paired two-tailed *t*-test using number of testes analysed as the *n* value).

During the course of our studies, we noticed that the elongated centrioles normally found in fly spermatocytes were significantly longer than normal when GFP–Ana1 was overexpressed, but not when GFP–Ana1ΔNT was overexpressed (Fig. 6A,B). The centrioles were also significantly shorter than normal in *ana1* heterozygous mutant spermatocytes (that have only one copy of the endogenous *ana1* gene) (Fig. 6A,B). We confirmed this result with four different spermatocyte centriole markers: Asl, GTU88* [a batch of an anti-γ-tubulin monoclonal antibody that cross reacts with spermatocyte centrioles (Martinez-Campos et al., 2004)], Spd-2 and Cep135. Interestingly, although the levels of Ana1 clearly influenced the length of spermatocyte centrioles, halving the dose of Ana1 did not lead to an obvious reduction in the levels of Cep135 or Asl localised at centrioles per unit length of centriole, whereas overexpressing GFP–Ana1 actually led to a slight decrease in the amount of Asl and Cep135 recruited to the centrioles per unit length (although this decrease was not statistically significant in the case of Cep135) (Fig. 6C). We conclude that Ana1 can promote centriole elongation in spermatocytes without recruiting more Cep135 or Asl (per unit length) to the centrioles.

**Fig. 7.**
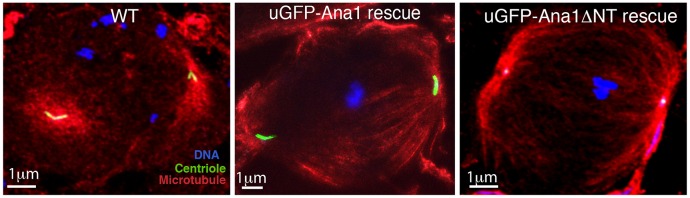
**GFP–Ana1ΔNT does not support centriole elongation in *ana1* mutant spermatocytes.** (A) Micrographs showing *Drosophila* mature primary spermatocytes in meiosis I stained for DNA (blue), microtubules (red) and the centriole marker Asl (green) in WT cells or *ana1* mutant cells overexpressing either GFP–Ana1 or GFP–Ana1ΔNT (as indicated). Centrioles are detectable at the poles of the spindles in the *ana1* mutant cells rescued by GFP–Ana1ΔNT, but these appear dot-like and have failed to elongate.

Although the centrioles in *ana1* mutant spermatocytes rescued by Ana1–GFP usually appeared to be abnormally elongated, those in mutant spermatocytes rescued by GFP–Ana1ΔNT were invariably extremely short (Fig. 7). These centrioles were so short that it was impossible to measure their length in immunofluorescence experiments as we could only discern them as dots, rather than elongated centrioles (Fig. 7; Fig. S2A, stage13). Nevertheless, the short centrioles in the *ana1* mutant spermatocytes rescued by GFP–Ana1ΔNT often went on to form short basal bodies at the base of the sperm nuclei, although these often appeared disorganised (Fig. S2A, stage 17), and mutant sperm were invariably immotile, presumably explaining why these flies are male sterile. Thus, although Ana1ΔNT can rescue the centrosome assembly defect in *ana1* mutant spermatocytes (Fig. S2B) and brain cells (Fig. 1B), it cannot support the dramatic elongation of the centrioles that normally occurs in spermatocytes.

**Fig. 8.**
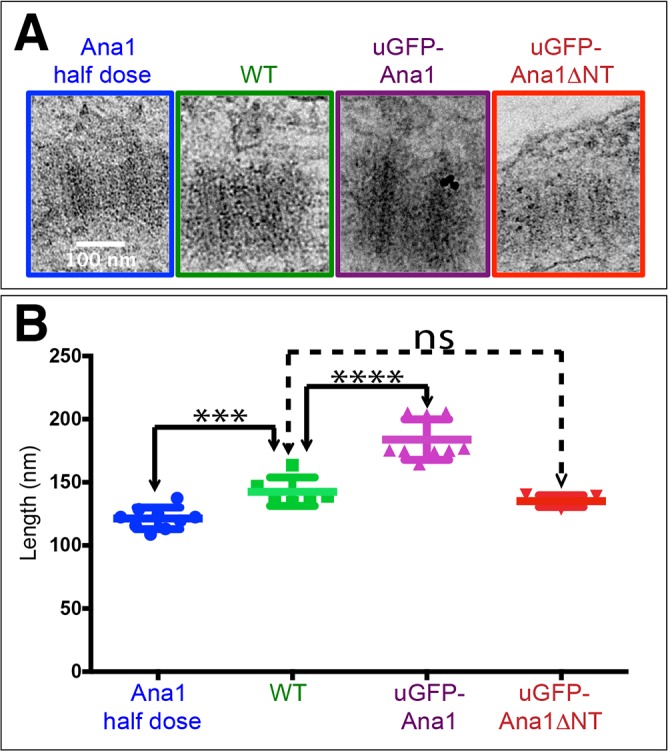
**Ana1 levels influence centriole length in wing disc cells.** (A) Electron micrographs showing images of centrioles viewed in longitudinal cross section in wing discs from *ana1* heterozygous mutants (containing only one copy of the WT gene), WT, WT overexpressing GFP–Ana1 and WT overexpressing GFP–Ana1ΔNT. (B) Graph quantifying the mean±s.d. centriole length (determined from electron microscopy images that were scored blindly) showing that the pattern of centriole length variation observed in spermatocyte centrioles (see Fig. 6) is also observed in wing disc centrioles. Each point on the graph represents the average centriole length of ten centrioles in an individual wing disc (nine, six, nine, and five wing discs were scored, respectively). ***P<0.0001, ****P<0.00001; ns, not significant (paired two-tailed *t*-test using number of wing discs analysed as the *n* value).

Because the centrioles in fly spermatocytes are much longer and more elaborate than those found in other fly tissues (Gonzalez et al., 1998), we examined centriole length by electron microscopy in fly wing disc cells, which contain centrioles more typical of those found in most *Drosophila* tissues. We found that GFP–Ana1 (but not GFP–Ana1ΔNT) overexpression led to an increase in centriole length, whereas halving the gene dosage of endogenous *ana1* led to a decrease in centriole length (Fig. 8A,B). Taken together, these observations demonstrate that Ana1 can promote centriole elongation in a dose-dependent manner, and that this activity appears to require the N-terminal 639 amino acids of Ana1.

## DISCUSSION

Our results are consistent with recent reports that Ana1/Cep295 is required for centriole-to-centrosome conversion in flies and humans (Fu et al., 2016; Izquierdo et al., 2014), and we show that *ana1* mutant cells have very few centrosomes *in vivo*. Studies on fly cells in culture suggested a simple model of centriole-to-centrosome conversion whereby Cep135 is initially recruited to centrioles and this subsequently recruits Ana1 to centrioles in late interphase. In mitosis, Ana1 then recruits Asl to new centrioles; Asl recruitment is crucial for centriole-to-centrosome conversion, as Asl is required to allow newly formed centrioles to recruit PCM during mitosis (Conduit et al., 2014b) and to duplicate during the next S-phase (Novak et al., 2014). A similar molecular mechanism appears to operate in human cultured cells (Fu et al., 2016).

Our findings suggest, however, that the recruitment of Ana1 to centrioles might be more complicated. The N-terminal region of Ana1 (amino acids 1–935) interacts with Cep135 *in vitro* and is recruited to centrioles in cultured cells, whereas an N-terminally deleted fragment of Ana1 (amino acids 756–1729) cannot be recruited to centrioles in cultured cells, even in the presence of the endogenous WT Ana1 protein. In contrast, we find that the C-terminal region of Ana1 can be recruited to centrosomes (although quite weakly). Moreover, GFP–Ana1ΔNT (comprising amino acids 640–1729) can rescue the centrosome assembly defect in *ana1* mutant cells and the uncoordinated phenotype of *ana1* mutant flies, although this protein is clearly not fully functional because it is only weakly recruited to centrosomes, and the rescued mutant flies are both male- and female-sterile. We do not know why this deletion construct appears to behave differently in embryos and cultured cells, but our findings suggest that several mechanisms help to localise Ana1 to centrioles in embryos. In particular, the middle region of Ana1 contains multiple predicted coiled-coil regions that could allow Ana1 to interact with itself (potentially complicating the analysis of protein localisation experiments performed in the presence of the WT endogenous protein) and also with other proteins such as Cep135.

Surprisingly, we also find that in the presence of GFP–Ana1ΔCT (a C-terminal Ana1 deletion that cannot interact with Asl) (Fu et al., 2016) the recruitment of Asl is more strongly reduced at old mother centrioles than at new mother centrioles. This is unexpected, and suggests that the process of recruiting Asl to mother centrioles might also be more complicated than originally thought. Indeed, mother centrioles appear to contain at least two pools of Asl: ∼50% of the Asl protein is stably incorporated into centrioles, whereas ∼50% is in exchange with the cytoplasmic pool (Novak et al., 2014). Interestingly, Asl interacts directly with another centriole protein, Sas-4/CPAP, in both fly and human cells (Cizmecioglu et al., 2010; Dzhindzhev et al., 2010; Hatch et al., 2010), and we have shown that Sas-4 plays an important part in recruiting Asl to new mother centrioles, but is not required to maintain Asl at old mother centrioles (Novak et al., 2014; Novak et al., 2016). Perhaps Sas-4 plays a more important role in initially recruiting Asl to new mother centrioles, whereas Ana1 plays a more important role in maintaining Asl at mother centrioles.

In human cells, centrioles are destabilised in the absence of Cep295. This appears to be because the failure in centriole-to-centrosome conversion blocks the recruitment of the PCM, and the PCM is required to stabilise the centrioles after they lose their central cartwheels during the conversion process (as normally occurs in vertebrates) (Izquierdo et al., 2014). In flies, however, the centrioles do not usually lose their cartwheels during the conversion process (Gonzalez et al., 1998), so it is unclear why centrioles appear to be destabilised in the absence of Ana1 (as the permanent central cartwheel structure would presumably stabilise centrioles even when they cannot recruit PCM). We observed that centriole-like structures can be seen at the electron microscopy level in *ana1* mutant cells overexpressing Sas-6 and Ana2 (Fig. 3B), and some Sas-4-containing structures are visible in immunofluorescence images from early *ana1* mutant spermatocytes (Fu et al., 2016), suggesting that some residual centriole-like structures can persist in *ana1* mutant cells. If some residual centriole structures do persist in *ana1* mutant tissues, however, they are clearly not capable of supporting centrosome assembly (Fig. 1B) or cilium function (as *ana1* mutant flies are severely uncoordinated).

We find that Ana1 promotes centriole elongation in a dose-dependent manner: centrioles are slightly longer when Ana1 is overexpressed, and slightly shorter when *ana1* gene dosage is halved. This finding is in contrast to the observation that Ana1 depletion does not lead to a decrease in centriole length in S2 cells (Fu et al., 2016), perhaps because Ana1 depletion does not alter the length of centrioles that had already formed in the cell population prior to Ana1 depletion (and our dynamic analysis of Ana1 behaviour suggests that Ana1 is irreversibly incorporated into centrioles). Importantly, this function of Ana1 appears to have different molecular requirements to the function of Ana1 in centriole stabilisation, as the N-terminal 639 amino acids of Ana1 are not essential for centrosome assembly or cilium function, but are required to allow Ana1 to promote centriole elongation in spermatocytes and to promote centriole over-elongation when overexpressed.

Several centriole proteins can influence centriole length. Positive regulators such as Sas-4/CPAP, Cep135, Cep120 and SPICE can increase centriole length when overexpressed in human cells, and negative regulators such as CP110, Cep97 and Klp10A appear to act to suppress centriole elongation (Comartin et al., 2013; Delgehyr et al., 2012; Franz et al., 2013; Kohlmaier et al., 2009; Lin et al., 2013a,b; Mahjoub et al., 2010; Schmidt et al., 2009; Tang et al., 2009). Although it is unclear how Ana1 influences centriole length, it is intriguing that its N-terminal region, which is required to promote centriole elongation, can interact with Cep135 (Fu et al., 2016) as centrioles are shorter than normal in *Cep135* mutant spermatocytes (Mottier-Pavie and Megraw, 2009). Our data suggests, however, that Ana1 does not promote centriole elongation simply by recruiting extra Cep135 or Asl to the centrioles. Interestingly, Cep295, the human homologue of Ana1, also promotes centriole elongation in human cells (suggesting that this function is conserved), and it can interact with tubulin –potentially providing a molecular mechanism that can explain how Cep295 promotes centriole elongation (Chang et al., 2016). To our knowledge, Ana1 is the first protein shown to reduce centriole length when its gene dosage is halved, suggesting that Ana1 is a limiting factor that ensures proper centriole elongation in flies.

## MATERIALS AND METHODS

### Fly stocks

The following fly lines were used in this study: OregonR flies (used as WT), flies expressing eAna1–GFP and eAsl–GFP at endogenous levels (Blachon et al., 2008), *ana1^mecB^* mutant flies (Avidor-Reiss et al., 2004), RFP–PACT-expressing flies (Lucas and Raff, 2007), flies expressing eAsl–mCherry at endogenous levels, and the *asl^B46^* mutant (Baumbach et al., 2015). The transgenic lines contain GFP fusions expressed from the Ubq promoter, which drives moderate expression in all tissues (Lee et al., 1988): GFP–Ana1 (WT), Ana1(WT)–GFP, Sas-6–GFP and Cep135–GFP (this study); GFP–Sas-6 (Peel et al., 2007); Asl–GFP, GFP–Ana2 and Ana2–GFP, and GFP–Ana1ΔNT (Stevens et al., 2010a) and GFP-Cep135 (Roque et al., 2012). The *Df(3R)Exel7357* that uncovers the *ana1* gene was obtained from the Bloomington *Drosophila* Stock Center (BDSC). Throughout this study flies or cells referred to as ‘*ana1* mutant’ have the genotype *ana1^mecB^/Df(3R)Exe7357*.

### Antibodies for immunofluorescence and western blotting

The following primary antibodies were used for immunofluorescence: rabbit anti-Asl (Conduit and Raff, 2010), guinea-pig anti-Asl (Roque et al., 2012), rat anti-Asl (Franz et al., 2013), mouse anti-GTU88* (Sigma-Aldrich), mouse anti-α-tubulin (DM1 α; Sigma-Aldrich), rabbit anti-Cnn (Lucas and Raff, 2007), sheep anti-Cnn (Conduit et al., 2014a), rabbit anti-Ana1CT (Stevens et al., 2010a), rabbit anti-Ana1Mid (Conduit and Raff, 2010), guinea-pig anti-Ana1 (Conduit et al., 2014b), rat anti-Ana1CT (this study), rabbit anti-Sas4 (Basto et al., 2006) and rabbit anti-Spd-2 (Dix and Raff, 2007) antibodies, all used at 1:500 (see details in Table S1). Secondary antibodies conjugated to Alexa Fluor 405, 488, 568, 594 (used for SIM) and 647 (Invitrogen) were used, all at 1:1000; GFP-booster–atto488 (ChromoTek) was used at 1:500. DNA was labelled with Hoechst 33342 (Invitrogen). Rabbit anti-Ana1 (Conduit et al., 2010) and mouse anti-actin (Sigma-Aldrich) antibodies, and appropriate horseradish peroxidase (HRP)-conjugated secondary antibodies (GE Healthcare) were used for western blotting (all 1:3000).

### Generation of transgenic lines

P-element-mediated transformation vectors containing GFP fusions to Ana1 were generated by cloning the genomic region of *ana1* from the start codon up to the stop codon into the Ubq-GFP(NT) or pUAST-GFP(NT), or without the stop codon into Ubq-GFP(CT) or pUAST-GFP(CT) Gateway vectors as described previously (Basto et al., 2008). Similarly, GFP fusions to Sas6 or Cep135 at their C-terminal were generated from their respective cDNAs using the Ubq-GFPCT or pUAST-GFPCT Gateway vectors (full cloning details are available upon request). Constructs were injected and transgenic lines were generated at Bestgene (USA) or at the Department of Genetics, University of Cambridge (UK).

### Immunofluorescence analysis of tissues

*Drosophila* brains from third-instar larvae or *Drosophila* testes from late pupae were dissected, fixed and stained as described previously (Franz et al., 2013). *Drosophila* eggs or embryos were collected, fixed and stained as described previously (Stevens et al., 2010a). Images were obtained on a confocal microscope system (Fluoview FV1000; Olympus) using a 60×1.4 NA oil objective and Fluoview software (Olympus), or on Zeiss Axioskop 2 microscope (Carl Zeiss, Ltd., UK) with a CoolSNAP HQ camera (Photometrics, Tucson, AZ) using a 63×1.25 NA objective (Zeiss) with Immersol oil (Zeiss) and MetaMorph software (Molecular Devices). All live-cell images acquired were analysed using Volocity software (Perkin Elmer). Quantification of centriole length in spermatocytes by assessing immunofluorescence images was performed as described previously (Franz et al., 2013). Quantification of Asl and Cep135 mean intensities per μm of centriole length in spermatocytes by assessing immunofluorescence images was performed in Fiji (ImageJ 1.48f) using the line tool (line width=9). Background fluorescence was subtracted before obtaining the final value. Quantification of SAP numbers per cell, SAP intensity and SAP area was performed using Volocity software (Perkin Elmer) by manually drawing a region of interest (ROI) around each SAP. Data was analysed in Prism (version 6.0 for Mac OSX, GraphPad Software, Inc).

### Live-cell imaging

Embryos (1–2-hours-old) were imaged, and individual centrosomes photobleached, on a Perkin Elmer ERS Spinning Disc confocal system on a Zeiss Axiovert microscope, using a 63×1.4 NA, oil-immersion objective. A total of 11 or 13 confocal sections were collected from each embryo (0.5-µm steps) every 20 or 30 s and were analysed using Volocity software (Perkin Elmer, USA) as described previously (Novak et al., 2014). Data was analysed and curve fitting performed in Prism (version 6.0 for Mac OSX, GraphPad Software, Inc).

### 3D-SIM and analysis

Preparation, fixation and staining of squashed late-pupal testes for 3D-SIM was as described previously (Franz et al., 2013). Images were taken at 21°C on an OMX-V3 system (GE Healthcare) with a 60×1.42 NA oil objective (Olympus) and SIM reconstruction was performed with SoftWorx software (Applied Precision). Different image channels were aligned using 200-nm TetraSpeck (Thermo Fisher Scientific) bead slides as reference for the transformation. Images shown are maximum intensity projections of several *z*-slices in the central area of the centrioles that were processed in Fiji (ImageJ 1.48f). GFP protein radial diameters were calculated by fitting a double-Gaussian curve to the average fluorescence intensity profile of each protein (obtained by drawing ten lines through each centriole and averaging the intensity profile of each line, as indicated by the white lines in the GFP–Ana1 panel in Fig. 4A); the distance between the two peaks was measured in Fiji (ImageJ 1.48f). Where it was not possible to resolve the two peaks, the diameters were measured by fitting the intensity profile crossing the centriole centre to two Gaussian curves of equal heights and standard deviations. The radial diameter calculated is the distance between the centre positions of each Gaussian peak. Analysis was performed with GNU Octave version 4.0.0 and Octave Forge optim package version 1.4.1. Data was analysed in Prism (version 6.0 for Mac OSX, GraphPad Software, Inc).

### Western blotting

Protein extracts from embryos, brains, wing discs and testes were separated on protein gels as described previously (Novak et al., 2014). In brief, samples containing ten embryos, two brains, four wing discs or two testes were run on NuPAGE 3–8% Tris-acetate pre-cast gels (Life Technologies). The proteins were transferred onto Biorad nitrocellulose membrane and loading was initially checked using Ponceau staining. The membrane was then blocked with 4% milk powder, 0.1% Tween-20 in PBS and probed with antibodies against Ana1, and against actin as a loading control.

### Transmission electron microscopy

Wing discs from third-instar larvae and testes from late pupae were dissected in PBS and prepared as described previously (Stevens et al., 2010b). Semi-thin serial sections (100 nm) were obtained by using a Reichert-Jung Ultracut E (Leica Microsystems, Austria) and stained in lead citrate. To measure centriole length in interphase cells from wing discs, images of centrioles in longitudinal orientation were taken on a TECNAI T12 transmission microscope (FEI, The Netherlands) at 11,000× or 18,500× magnification. The length of the microtubule doublets within the electron-dense area was measured using the line tool in Fiji (ImageJ 1.48f). Data were analysed in Prism (version 6.0 for Mac OSX, GraphPad Software, Inc). For measurement of the number of testes SAPs, semi-thick serial sections (150 nm) were obtained and dual-axis tilt series (typically −55°–55°) were acquired in a TECNAI T12 transmission microscope (FEI) at 13,000× magnification with SerialEM. Using the IMOD software package (Mastronarde, 2005) the images were aligned, and the tomograms were reconstructed.

### *In vitro* mRNA production and injection

The complete coding region of Ana1 protein was amplified from cDNA IP16240 (DGRC) with att sites at either end, and inserted into the Gateway pDONR-Zeo vector (Invitrogen). Ana1 deletions were generated by site-directed mutagenesis of pDonor-Zeo-Ana1 (after repairing the missing 67 bp from the available Ana1 cDNA), using the Quikchange II XL mutagenesis kit (Agilent). Deletions were cloned in Gateway vectors (Invitrogen) into the pRNAGFP destination vector (Conduit et al., 2014a). WT or truncated versions of Ana1 fused to an N-terminal GFP mRNA were synthesised and injected into early embryos expressing eAsl–mCherry as described previously (Cottee et al., 2013; Richens et al., 2015). Syncytial stage embryos were imaged on a Perkin Elmer microscope and analysed using Volocity software (Perkin Elmer) as described previously (Novak et al., 2014). Briefly, we measured the total GFP or mCherry fluorescence in a boxed region centred around the centrosomes and subtracted the local cytoplasmic background fluorescence (*n*=10 centrosomes from five embryos for each construct). Each value was normalised to the averaged WT signal (set to one). The Fluorescence values were compared using a *t*-test (unpaired). Data was analysed in Prism (version 6.0 for Mac OSX, GraphPad Software, Inc).
